# Predictors of Patient Satisfaction and the Perceived Quality of Healthcare in an Emergency Department in Portugal

**DOI:** 10.5811/westjem.2019.9.44667

**Published:** 2020-01-27

**Authors:** Alina Abidova, Pedro Alcântara da Silva, Sérgio Moreira

**Affiliations:** * NOVA University of Lisbon, National School of Public Health, Lisbon, Portugal; † University of Lisbon, Institute of Social Sciences, Lisbon, Portugal; ‡ University of Lisbon, Faculty of Psychology, Lisbon, Portugal

## Abstract

**Introduction:**

The predictors of patient satisfaction in emergency medicine (EM) have been widely studied and discussed in the scientific literature; the results vary depending on the specific EM attributes, cultural aspects, researchers’ preferences, and approaches. However, it is not clear whether the same predictors of patient satisfaction can contribute to a better-perceived quality of healthcare or whether patients’ perceptions form a different attitude toward satisfaction and perceived quality of healthcare. The goal of this study was to identify the key predictors of patient satisfaction and perceived quality of healthcare in the framework of an emergency department (ED).

**Methods:**

We conducted a retrospective study of patients seen at an ED between January –December 2016. Data collection took place in the public hospital in Lisbon, Portugal, between May – November 2017. The total sample size included 382 patients. The sample distribution had a 5% margin of error and a 95% confidence interval. Data for this research, using a questionnaire, was collected by mail or e-mail according to the respondent’s preference.

**Results:**

A detailed analysis showed that three out of the 18 predictors had a statistically significant relationship with satisfaction: overall satisfaction with doctors, with a positive correlation (r = 0.14, p ≤ 0.01); qualitative perceived waiting time for triage, with a positive correlation (r = 0.08, p ≤ 0.05); and meeting expectations, with a positive correlation (r = 0.53, p ≤ 0.01). Furthermore, a detailed analysis showed that only two out of the 18 predictors had a statistically significant relationship with the perceived quality of healthcare (PQHC): overall satisfaction with doctors, with a positive correlation (r = 0.43, p ≤ 0.01) and meeting expectations, with a positive correlation (r = 0.26, p ≤ 0.01).

**Conclusion:**

The main predictors of satisfaction and perceived quality of healthcare were overall satisfaction with doctors and meeting expectations. We should note that “meeting expectations” plays the most important role in terms of satisfaction; however, in terms of PQHC the predictor “overall satisfaction with doctors” plays the most important role due to its stronger correlation. In addition, the qualitative perceived waiting time for triage could be considered as another predictor, influencing satisfaction only, thus emphasizing similarities and differences between satisfaction and the PQHC in an ED context.

## INTRODUCTION

Patient satisfaction plays a crucial role in the healthcare system as an indicator of the quality of care.^1^ Importantly, the patient’s experience of care is increasingly being used to determine hospital and physician reimbursements.^2^ In this respect, patient satisfaction is subject to monitoring and assessment on an individual, community, and regional scale. The predictors of patient satisfaction in an emergency department (ED) are widely studied and discussed in the scientific literature, where the primary focus is ED staff. It is generally accepted that good nursing care as well as friendly and attentive staff members are of high importance for patients when visiting the ED.^3^,^4^,^5^ Patient dissatisfaction with the ED encounter is frequently related to poor communication.^6^,^7^ The physician-patient relationship, built upon verbal and non-verbal communication, is particularly important in EDs.^7^,^8^,^9^ However, it is not clear whether the same predictors of patient satisfaction could contribute to a better-perceived quality of healthcare or whether patient perceptions could form a different attitude toward satisfaction and the perceived quality of healthcare.

Patient experience measures have been shown to be indicators of healthcare quality; at the same time, there is no common approach for defining “patient satisfaction.”^10^ Patient satisfaction is measured through patient experiences with the healthcare system, which allows researchers, industry professionals, and policymakers to identify problems and outline areas for improvement to ensure equity in access and the availability of care services.^11^ The main aim of measuring patient experience and satisfaction is to understand how the patient feels about being treated, learn about his/her perceptions of the quality of care and any related constructs, and to highlight areas of practice that could be improved to achieve better health outcomes and patient loyalty.^12^

One of the important parameters of patient satisfaction with the ED is based on how patients select a particular ED and whether they would recommend it to other patients.^13^ Such important metrics contain patients’ viewpoints and expectations, which are necessary to improve the quality of healthcare services. However, the relationship between expectation and satisfaction is unclear.^14^ Since healthcare is targeted at patients, it is only natural that their expectations and ideas be incorporated into the delivery of healthcare services. Patient satisfaction with the ED may be influenced by numerous factors, including experience with nursing care, communication, infrastructure, and environment in which the healthcare professional practices.^9^,^15^ Patient factors that may influence satisfaction include age, gender, income, education level, expectations, marital status, and where they live.^13^ Hospital-related factors such as staff, waiting times, facilities, and processes may also influence patient satisfaction.^16^ Hence, satisfaction is a widely measured concept that is not easy to define; however, it still needs to be developed.^14^,^17^

Patient satisfaction is related to the quality of care provided, and correlation between these two constructs highlights the need for collecting opinions regarding the care provided by the healthcare system.^18^,^19^ Collecting patients’ perceptions of quality of care is indispensable to attain crucial insight into their experiences, views, and opinions about hospital wards. What quality of care means is different depending on the different stakeholders. The Institute of Medicine’s “Crossing the Quality Chasm”^20^ provides a framework for defining the quality of healthcare. It provides guidelines to evaluate and determine the quality of healthcare delivery. The report conceptualizes the quality of care in six dimensions: safety; efficiency; effectiveness; timeliness; equity; and patient-centeredness.^20^

The World Health Organization^21^ associates the quality of healthcare with six dimensions: effectiveness; efficiency; accessibility; acceptable/patient-centered care; equitability; and safety. The determinants of the quality of care include patient factors, technical quality, the quality of interpersonal interactions, and clinical factors.^16^ Communities and service users, health service providers, and policy and strategy developers all have roles and responsibilities to ensure the delivery of quality healthcare.^21^ Therefore, it is necessary to distinguish between satisfaction and the perceived quality of healthcare. It is also important to understand whether the same or a different set of factors could contribute to their improvement in the ED. Our main goal was to identify the key factors promoting patient satisfaction and perceived quality of healthcare in the ED including the following: 1) expectations (meeting expectations); 2) global perceptions (accessibility, availability; facilities, physical conditions; privacy; busyness of the ED in terms of number of people); and 3) perceived quality dimensions (ED staff; agreement with color assigned (triage level); waiting times; and information about possible delays).

**Population Health Research Capsule**

What do we already know about this issue?

Patient satisfaction and perceived quality of healthcare (PQHC) are used as measures of the evaluation of patients’ experiences and perceptions in the emergency department.

What was the research question?

What are the key predictors of patient satisfaction and the PQHC, and do these or a different set of factors contribute to perception of quality of care in the ED?

What was the major finding of the study?

Patient satisfaction and PQHC have two key predictors in common, overall satisfaction with doctors and meeting expectations.

How does this improve population health?

Patient satisfaction is a more important measure than PQHC, influenced by a larger number of factors while at the same time sharing some similarities with PQHC.

## METHODS

Data collection was carried out from May 18 – November 30, 2017, in the Hospital de São Francisco Xavier, the public hospital in Lisbon, Portugal. All responders were at least 18 years old, able to answer the questions, residents of Portugal, and Portuguese-speaking. We excluded respondents who were unable to answer the questions, who resided outside Portugal, or had psychiatric illnesses. Probability sample with a 5% margin of error and a 95% confidence interval was examined. The total sample size was 382 patients. To calculate our random probabilistic sample size, we used a list of 55,903 patients who entered the ED (January 1 – December 31, 2016) at least once at the public hospital. Before sending the questionnaire, all patients were contacted by telephone to obtain permission to send the questionnaire and consent to participate in the survey.

When a chosen individual had more than one ED admission in the year under study, we chose the last admission according to the date of admission. Telephone calls were made three times during the day at different times and if our attempts to reach him or her were unsuccessful, the patient was classified as not responsive. The questionnaire was sent either by mail or e-mail, depending on the respondent’s preference. If regular mail service was used the questionnaire was sent to the home address with an enclosed prepaid envelope. In cases of e-mail distribution, we used Qualtrics software (Qualtrics XM, Provo, UT/Seattle, WA) to collect the data online. During the data collection period we made a total of 4,413 telephone calls, just including the first-call attempts and excluding all repeat calls afterwards. Those who did not have a telephone number in our list were excluded prior to the initiation of the calls. In total, 2,512 (56.9%) individuals agreed to participate in the survey. Among the remaining 1,901 (43.1%) who did not participate 333 (7.5%) individuals declined to participate due to various reasons or simply did not want to participate in the survey; 157 (3.6%) individuals had already died, and 43 (1.0%) were ineligible per the exclusion criteria, as the phone was answered by another person. A total of 1368 (31.0%) individuals either did not respond to the telephone call, or had unassigned, invalid, temporarily disconnected, or incomplete phone numbers. Eventually, 1,553 patients agreed to participate and gave permission to us to send the questionnaire by mail; however, only 506 questionnaires were sent due to the study’s financial constraints. We received 143 (9.2%) responses to our questionnaires, and 363 (23.4%) did not respond. With respect to the e-mail distribution, 959 patients agreed to participate and gave us permission to send the questionnaire by e-mail. Of those email recipients, 340 (35.5%) responded to the questionnaire online, and 619 (64.5%) did not respond. Those individuals who did not respond and did not send back the questionnaire were contacted again and asked to complete it. In the case of an incorrect home address, the respondent was contacted again and then sent the questionnaire. The same was done with e-mail distribution; after a certain period of time the respondent was contacted again through e-mail and asked to respond to the online questionnaire. The total number of obtained questionnaires (483) exceeded the total number of a calculated necessary sample size (382), resulting in exclusion of 101 incomplete/poorly completed questionnaires where the number of questions answered was very low, as well as questionnaires that were returned after our data analysis had already begun. Thus, among the 382 individuals, 75.9% were online (e-mail) respondents, and 24.1% responded via regular mail.

Our modified-elaborated questionnaire was partly based on the questionnaire used by Pereira et al.^22^ and was partly based on the *Instrumentos de Avaliacao da Qualidade Hospitalar – Urgencias Adultos* [Portug.][Instruments for Evaluating Hospital Quality - Adult Emergency], which was designed, developed, and tested by the Centro de Estudos e Investigacao em Saude da Universidade de Coimbra [Portug.] [Center for Studies and Research in Health of the University of Coimbra].^23^,^24^ In addition, we took into consideration the fourth national health survey (Portugal) prepared by the Instituto Nacional de Saude Dr. Ricardo Jorge/Instituto Nacional de Estatistica [Portug.] [National Institute of Health Dr. Ricardo Jorge/National Institute of Statistics],^25^ as well as the survey used to investigate the aging process in Portugal.^26^

Variables that measured more than one item were simplified into a single composite measure. This was the case with the set of eight variables: 1) accessibility and availability; 2) facilities and physical conditions; 3) satisfaction with staff at the registration counter; 4) with personnel, conducting the triage; 5) with doctors; 6) with nurses; 7) with auxiliary staff; 8) and with health technicians responsible for examinations and/or tests. Accessibility and availability consisted of five items: 1) location; 2) orientation; 3) distance between the different areas; 4) availability of equipment and of specialist staff; and 5) overall satisfaction with accessibility and availability.

Facilities and physical conditions consisted of six items related to the condition, comfort, and convenience of the following areas: 1) the waiting room; 2) the observation room; 3) the facilities where tests were carried out; as well as 4) age and operation of equipment; 5) cleanliness and hygiene of the facilities; and 6) overall satisfaction with facilities and physical conditions. Patient satisfaction with staff at the registration counter, with personnel conducting the triage, nurses, auxiliary staff, with health technicians responsible for examinations and/or tests consisted of three items: 1) friendliness and helpfulness; 2) competence and professionalism; and 3) overall performance. Satisfaction with doctors consisted of six items: 1) friendliness and helpfulness; 2) competence and professionalism; 3) the way the doctor explained a health problem (diagnosis); 4) explanations given by the doctor on the exams performed and the objectives of the treatment to be undergone; 5) information provided on precautions to be taken, recommendations, and how to take or apply the medications prescribed; and 6) overall performance.

We used an exploratory factorial analysis (EFA) to test for the items’ underlying factors. The EFA was conducted using the principal axis factoring method for extraction, the scree plot for selecting the number of factors, and the oblimin rotation to interpret the factor loadings. We used a factor analysis to model the inter-relationships between multiple items but with fewer variables, to reduce composite scale variables with several measures into one single scale.^27^ Factor loading expressed the association of the variables to their underlying factors. The statistical significance of factor loadings was based on their magnitude.^27^ For the rotated factor loading for a sample of at least 300 participants to be statistically significant at an alpha level of 0.01 (two-tailed), it would need to be greater or equal to 0.32.^28^ In turn, we considered factor loadings above 0.30 to be acceptable, being statistically significant at 382 participants. All items used could be aggregated into single factors due to the strong correlations observed. More specifically, high alpha coefficients (0.87 to 0.99) evidence that the items have a relatively good internal consistency,^27^ consequently giving us confidence that our measures were reliable and correct.

## RESULTS

### Descriptive Analysis of Patient Satisfaction and Perceived Quality of Healthcare

The participants were mostly from Lisbon (96%) and were grouped into persons with dual nationality (2.1%), other nationality (2.6%), and Portuguese (95.3%), with the proportion of females to males at 61.3%: 38.7%. The age distribution of participants across age groups was almost uniform: 18–30 years (14.9%), 31–40 (19.1%), 41–50 (14.4%); 51–60 (17.6%); 61–70 (9.2%); 71–80 (9.8%); 80+ y (14.7%). The mean values, standard deviation, and correlation coefficients with two main variables, including descriptive statistics of the variables are shown in Tables 1 and 2.

The results show that two core variables of this study, satisfaction and PQHC, are strongly correlated (r = 0.80). Considering the possible correlations between satisfaction, PQHC, and other variables we were able to evidence that even though satisfaction and PQHC are very close concepts, they still differ. The data presented in Tables 1 Table 2 show the differences between satisfaction and the PQHC. Furthermore, the data demonstrate the different degree of correlation between the variables (moderate vs strong) in terms of satisfaction and PQHC, variables that disunite satisfaction and PQHC according to inclusion criteria (weak vs very weak correlation), and variables that unite satisfaction and PQHC.

Regarding satisfaction and PQHC, 24 variables appear to unite them, as compared to two variables that separate them. These two variables slightly differ in terms of the patients’ views. Agreement with the triage color assigned, for example, can be perceived as a more relevant issue in terms of satisfaction (r = 0.20), but slightly less relevant (r = 0.17) in terms of the PQHC. On the contrary, other variable such as a discharge note given to a patient (r = 0.20 vs r = 0.16) was slightly more relevant in terms of PQHC than in terms of satisfaction. An additional three variables – nursing personnel; evaluation of the treatment received; and evaluation of communication with relatives or with the people accompanying them about their health situation – showed a slightly different degree of correlation (moderate vs strong) in terms of satisfaction and the PQHC. With reference to ED personnel, patients relate nursing staff to PQHC (r = 0.61 vs r = 0.58) as being more relevant than satisfaction. Similarly, the evaluation of communication with relatives or with the people accompanying the patient about his or her health situation (r = 0.70 vs r = 0.47) and the evaluation of the treatment received (r = 0.66 VS r = 0.57) appear to be more relevant regarding the PQHC.

In terms of the waiting time variables (waiting time for triage; waiting time after triage; waiting time for examinations and/or tests; waiting time to be called back by the doctor after the examinations and/or tests; discharge waiting time), we analyzed the qualitative perceived waiting time (on a scale of 1–10) and the quantitative perceived waiting time (with an exact time scale evaluation). For example, waiting time for triage was measured both using a 1–10 scale and an exact time scale evaluation. The same was done with all other waiting time variables. It is important to manage the qualitative perceptions of waiting times, as different patients may perceive the same waiting time interval in a different way that may lead to contradictory results. Thus, our data show that qualitative perceived waiting times (on a 1–10 scale) have a stronger correlation with satisfaction and PQHC than quantitative perceived waiting times (with an exact time scale evaluation), represented in Tables 1 and 2.

Overall, it appears that the potential predictors correlate with satisfaction and the PQHC, among which some of them have stronger correlations than others, with either satisfaction or the PQHC. It suggests that, although being similar constructs, different predictors might explain them.

### Predictors of Patient Satisfaction and Perceived Quality of Healthcare

We applied a multiple regression analysis to identify the main predictors of satisfaction and the PQHC. Two important issues were examined: 1) how much the selected predictors account for satisfaction and the PQHC; and 2) which predictors stand out and how they differ between satisfaction and the PQHC. As expected, the qualitative perceived waiting time appeared to be the major predictor of satisfaction and the PQHC due to its stronger correlation level (Tables 1 and 2). In addition, other potentially relevant variables were excluded from the regression analysis due to extensive missing values (at least 30% of the total participants) among which were nursing personnel, auxiliary staff, evaluation of the treatment received, and evaluation of communication with relatives or with the people accompanying the patient about the health situation. We should note that the missing values in these variables result from the fact that not all the participants had contact with nursing personnel or auxiliary staff, received treatment, or were accompanied by a relative or another person. The benefits of still including these variables with missing values to have a more extensive list of the predictors did not justify the costs of having a reduced sample size and, consequently, reducing the test power for the study of the predictor.

Finally, only variables with a strong, moderate, or weak correlation with satisfaction and the PQHC were taken into consideration. Two regression models were computed, including the 18 selected predictors and including either satisfaction or PQHC as the dependent variables. We used the forced entry method (all predictors entering simultaneously into the regression model) as there were no specific predictions about the relative contributions of each variable (or block of variables).

The regression model with satisfaction shows statistically significant results (Table 3): F(18,234) = 45.49, adjusted R square = 0.76, and p ≤ 0.01. A more detailed analysis shows that three out of the 18 predictors have a statistically significant relation with satisfaction: *overall satisfaction with doctors*, with a positive correlation (r = 0.14, p ≤ 0.01); *qualitative perceived waiting time for triage*, with a positive correlation (r = 0.08, p ≤ 0.05); and *meeting expectations*, with a positive correlation (r = 0.53, p ≤ 0.01).

The regression model with the PQHC also showed statistically significant results (Table 4): F(18,248) = 33.97, adjusted R square = 0.69, and p ≤ 0.01. In the given case, the results show that only two out of the 18 predictors have a statistically significant relationship with the PQHC: *overall satisfaction with doctors*, with a positive correlation (r = 0.43, p ≤ 0.01) and *meeting expectations*, with a positive correlation (r = 0.26, p ≤ 0.01). Consequently, it appeared that *overall satisfaction with doctors* and *meeting expectations* could be the main predictors of satisfaction and the PQHC, while *qualitative perceived waiting time for triage* could be considered as another relevant predictor, but only in terms of satisfaction.

## DISCUSSION

In the first definition from the year 1975, patient satisfaction referred to “the degree of congruence between a patient’s expectation of the ideal care they receive.”^29^ A growing body of literature has focused on determining the value of obtaining patient expectations in a written format prior to receiving care in the ED.^30^ In turn, unmet expectations can result in patients’ non-compliance and may impact the providers’ reputation in a community; an estimated 70% of litigation involving medical practitioners can be related to real or perceived problems in communication, which influence patients’ expectations.^31^ Indeed, in our analysis, meeting patients’ expectations turned out to be among the main predictors of satisfaction and the PQHC. A strong correlation between two core variables, ie, satisfaction and PQHC, united in our study by 24 variables, further supporting the close similarity of these two concepts. However, some of the variables have stronger or weaker correlations to others, with either satisfaction or the PQHC demonstrating the subtle differences of these two core variables. It suggests that although being similar constructs, different predictors might explain satisfaction and the PQHC.

Patient satisfaction is identified as one of the most important goals in any ED, relying on patient-reported experience measures (PREM), which gains increasing attention as an indicator of the quality of health care.^32^ According to a recent systematic review, currently available PREMs for use in EDs have uncertain validity, reliability, and responsiveness.^33^ Several attempts to upgrade the validity of PREM have been explored. PREMs differ from patient-reported outcome measures, which aim to measure the patient’s health status quality, as well as more subjective patient satisfaction measures.^32^ According to our analysis, both satisfaction and PQHC appear to be subjective concepts, influenced by subjective measures, where patients tend to emphasize the importance of the same/various predictors at a different level in terms of satisfaction and PQHC that leads to distinction between them. Thus, we may observe a different level of correlation that proves that patients may form different views regarding these two concepts, even though observing their similarity at the same time.

It is important to give patients time to deliberate over their experience, forming a true point of view. In prior research on access to, evaluation of, and attitudes toward the health system in the Portuguese population, it was shown that the memory of the hospital experience is valid up to three years, depending on the type of services and care received. In these studies, the experience in the ED was shown to be recalled for up to three years (last experience), which supports our temporal option about the research period.^34^,^35^,^36^ The decision to cover a full year aimed to take into account the effects of seasonality, which affects the use of emergency services and the type and incidence of different illnesses. For example, when a patient’s satisfaction is measured one hour after a single treatment in the ED, it does not capture a patient’s view of their entire visit.^37^ Healthcare service quality indicators, including health providers’ interpersonal care, are repeatedly the most influential determinants of patient satisfaction.^38^

Some researchers have pointed to an important role of nurses in the ED. The role of nurses in the ED influences the quality of care because the early recognition and addressing of symptoms can determine the quality of patient outcomes.^39^,^40^ Nursing care, including care and concern, keeping patients informed about delays, technical skills, keeping family and friends informed extend the role of nursing staff and were significantly associated with patient satisfaction.^39^ Nursing personnel in our study were excluded from the regression analysis due to extensive missing values, even though we observed a strong correlation with the PQHC.

According to the results from the regression analysis, overall satisfaction with doctors came to the fore among the main predictors of satisfaction and PQHC that incorporated several items: friendliness and helpfulness; competence; and professionalism. Among the other important items were the way the doctor explained a health problem (diagnosis), explanations given by the doctor on the exams performed and the objectives of the treatment to be undergone, the information provided on the precautions to be taken, recommendations and how to take or apply the medications prescribed, and the overall performance. Physician care and concerns expressed, giving advice and follow-up, the accuracy of explanations regarding the treatment and tests, and keeping the patient informed; all these items were strong predictors of overall patient satisfaction.^39^ The high importance of the doctor-patient relationship and communication, which can influence patient satisfaction, has been pointed out by several reserachers.^41^ Patients placed a high importance on the use of plain language by a doctor (the way the patient understands) (92.1%), and the explanations given during each step of examination (90.8%).^42^ Consequently, observing different attributes incorporated into the doctors’ notion, our results are consistent with other results from the literature.^39^,^41^,^42^

Another major predictor of satisfaction identified in our analysis was the qualitative perceived waiting time for triage. This time factor may vary across EDs, hospitals, regions, and even countries, depending on the efficiency of the ED and healthcare system. Several researchers investigated waiting time for triage in the ED and patient satisfaction resulting from a color assigned in triage.^43^ Our results confirmed the importance of this waiting time having a significant relationship with overall satisfaction.

By understanding that the essence of the main predictors of patient satisfaction is the importance of communicating with patients it will become clearer how providers can identify ways to improve their interactions with patients.. Prioritizing fulfillment of medical functions, ED clinical staff may ignore spending time on interacting with patients since approximately 75% of a patient’s time in a care area is spent not interacting with care providers.^44^ Neglected communication may cause acute problems in emergency medicine since 12% of errors are attributed to communication problems.^45^ Continuous overload and exposure to physical suffering reduce the staff’s susceptibility to the emotional needs of acute care patients.^46^ Several researchers have emphasized the importance of communication in the ED context that may influence the experience of waiting time as well as the importance of the responsiveness of staff that capture patient satisfaction.^47^,^48^,^49^ In the context of waiting times, the absence of physician or nurse attention forms the overall perception of ED care.^17^ In the pursuit of patient satisfaction, physicians and nurses modify their clinical and communication practices boosting an improvement in the quality of care.^50^

## LIMITATIONS

Our data collection was subject to some limitations as it was confined to one ED in one country. In addition, we took into consideration only the Portuguese-speaking population and those who were able to answer the questions, which further reduces the generalizability of our findings. We chose the sample distribution with a 5% margin of error rather than a lower margin of error due to time and financial constraints. A longitudinal study would be a preferable choice, as some of the effects may present temporal lags.

## CONCLUSION

Several patient- and hospital-level predictors can be consistently associated with patient satisfaction where patient-centered communication plays a vital role. Our study confirmed that overall satisfaction with doctors and meeting expectations are the main predictors that influence satisfaction and the PQHC. We should note that meeting expectations plays the most important role in terms of satisfaction; however, in terms of PQHC the most important factor is overall satisfaction with doctors due to its stronger correlation. Qualitative perceived waiting time for triage is considered to be another predictor that will influence only satisfaction, thus emphasizing similarities and differences between satisfaction and the PQHC in an ED context.

## Figures and Tables

**Table 1:** Means, minimum, maximum, standard deviations, and correlations with satisfaction and the Perceived Quality of Healthcare.

	n	Mean	Minimum	Maximum	SD	r_Satisfaction_	r_Quality_
Age (years)	382	53.19	20	92	20.235	0.20	0.21
Accessibility and availability						0.65	0.63
Location of the hospital and emergency department within the city	379	8.20	1	10	1.96	-	-
Orientation within the emergency department	374	7.44	1	10	2.05	-	-
Distance between the different areas of the emergency department	363	7.46	1	10	1.92	-	-
Availability of equipment and of specialist staff to conduct tests, blood tests	366	7.32	1	10	2.19	-	-
Overall, accessibility, and availability	375	7.49	1	10	2.08	-	-
Facilities and physical conditions						0.63	0.60
Conditions, comfort, and convenience of the waiting room	371	5.07	1	10	2.43	-	-
Conditions, comfort, and convenience of the observation room	379	6.17	1	10	2.31	-	-
Conditions, comfort, and convenience of the facilities where tests were carried out	363	6.68	1	10	2.15	-	-
Age and operation of equipment	339	6.81	1	10	2.06	-	-
Cleanliness and hygiene of the facilities	377	6.72	1	10	2.37	-	-
Overall, the facilities, and physical conditions of the emergency department	376	6.48	1	10	2.13	-	-
Privacy						0.45	0.46
The way the privacy was safeguarded	372	7.27	1	10	2.41	-	-
Staff at the registration counter						0.54	0.51
Friendliness and helpfulness of staff at the registration counter	371	7.22	1	10	2.22	-	-
Competence and professionalism of staff at the registration counter	368	7.40	1	10	2.15	-	-
Overall, the performance of the staff	372	7.46	1	10	2.13	-	-
Waiting time for triage (perception)						0.47	0.40
Waiting time for triage in view of the severity of condition	362	7.35	1	10	2.37	-	-
Staff conducting the triage						0.51	0.52
Friendliness and helpfulness of the nurse conducting the triage	367	7.73	1	10	1.99	-	-
Competence and professionalism of the nurse conducting the triage	366	7.82	1	10	1.94	-	-
Overall, the performance of the nurse conducting the triage	366	7.84	1	10	1.92	-	-
Waiting time after triage (perception)						0.55	0.43
Waiting time to be seen by a doctor after the triage in view of the severity of the condition	372	5.21	1	10	2.98	-	-
Doctors						0.65	0.76
Friendliness and helpfulness of the doctor(s)	379	7.74	1	10	2.17	-	-
Competence and professionalism of the doctor(s)	374	7.90	1	10	2.15	-	-
The way the doctor explained a health problem (diagnosis) during the examination	378	7.78	1	10	2.30	-	-
The explanations given by the doctor on the exams performed and the objectives of the treatment to be undertaken	366	7.77	1	10	2.39	-	-
The information provided on precautions to be taken, recommendations, and how to take or apply the medications prescribed (written or oral) after leaving hospital	370	7.95	1	10	2.23	-	-
Overall, the performance of the doctor(s)	378	7.89	1	10	2.26	-	-
Nursing personnel						0.58	0.61
Friendliness and helpfulness of the nurses	258	8.05	1	10	1.93	-	-
Competence and professionalism of the nurses	256	8.22	1	10	1.87	-	-
Overall, the performance of the nurses	260	8.20	1	10	1.92	-	-
Auxiliary staff						0.44	0.51
Friendliness and helpfulness of the auxiliaries	123	8.17	1	10	1.89	-	-
Competence and professionalism of the auxiliaries	121	8.17	1	10	1.76	-	-
Overall, the performance of the auxiliary staff	122	8.26	1	10	1.78	-	-
Waiting time for examinations and/or tests (perception)						0.58	0.54
Waiting time for examinations and/or tests in view of the severity of the condition	311	5.98	1	10	2.66	-	-
Waiting time to be called back by the doctor (perception)						0.59	0.57
Waiting time to be called back by the doctor after the examinations and/or tests in view of the severity of the condition	314	5.58	1	10	2.71	-	-
Health technicians						0.58	0.59
Friendliness and helpfulness of the health technicians in question	322	7.52	1	10	2.04	-	-
Competence and professionalism of the health technicians in question	312	7.77	1	10	1.99	-	-
Overall, the quality of the services provided with examinations or tests	319	7.72	1	10	1.94	-	-
Evaluation of the treatment received						0.57	0.66
Evaluation of the treatment received	224	8.24	1	10	1.90	-	-
Evaluation of communication with relatives or with the people accompanying the patient about health situation						0.47	0.70
The way the emergency physician or nurse communicated with relatives or with the people accompanying about health situation	164	8.30	1	10	1.79	-	-
Discharge waiting time (perception)						0.44	0.43
Waiting time from when the patient was informed about discharge until the patient left the hospital	317	7.67	1	10	2.60	-	-
Expectations						0.83	0.70
Meeting the expectations	375	6.65	1	10	2.39	-	-
Satisfaction							0.80
Considering the entire experience at the ED, the level of satisfaction	380	7.10	1	10	2.38		
Perceived quality of healthcare						0.80	
Overall, evaluation of the quality of healthcare	373	7.65	1	10	2.10		

**Table 2:** Total number, percentage, and correlations with satisfaction and Perceived Quality of Healthcare.

	n	%	r_Satisfaction_	r_Quality_
Lack of any type of staff			−0.37	−0.30
You did not feel the need for any type of staff	120	31.4	-	-
Doctors	148	38.7	-	-
Nurses	95	24.9		
Auxiliaries (for example, those bringing food, moving stretchers, accompanying patients, etc.)	81	21.2	-	-
Health technicians (conducting tests)	50	13.1	-	-
Administrative staff	12	3.1	-	-
Busyness of the emergency department, in terms of number of people (users/patients)			−0.27	−0.21
Not very busy	21	5.6	-	-
Normal number of people	110	29.6	-	-
Very busy	147	39.5	-	-
Too busy	94	25.3	-	-
Total	372	100.0	-	-
Information about possible delays in receiving treatment or waiting times			0.24	0.21
Yes	59	16.6	-	-
No	297	83.4	-	-
Total	356	100.0	-	-
Explanations for the delay			0.39	0.33
Yes	24	6.7	-	-
No	235	65.8	-	-
I did not wait for a long time	98	27.5	-	-
Total	357	100.0	-	-
Agreement with (triage) color assigned			0.20	0.17
Yes, I agreed with the color assigned	225	75.5	-	-
No, I should have been assigned a more urgent color	73	24.5	-	-
Total	298	100.0	-	-
If the patient was given a discharge note (letter summarizing what happened in the emergency department)			0.16	0.20
Yes	265	75.7	-	-
No	85	24.3	-	-
Total	350	100.0	-	-
Waiting time for triage			−0.25	−0.22
No waiting period	46	12.6	-	-
Up to 5 minutes	110	30.1	-	-
Over 5 and up to 15 minutes	114	31.1	-	-
Over 15 and up to 30 minutes	49	13.4	-	-
Over 30 minutes up to 1 hour	25	6.8	-	-
Over 1 hour	22	6.0	-	-
Total	366	100.0	-	-
Waiting time to be seen by a doctor after the triage			−0.35	−0.31
No waiting period	20	5.6	-	-
Up to 15 minutes	47	13.1	-	-
Over 15 and up to 30 minutes	57	15.9	-	-
Over 30 minutes and up to 1 hour	76	21.2	-	-
Over 1 and up to 2 hours	69	19.2	-	-
Over 2 and up to 4 hours	61	17.0	-	-
Over 4 and up to 6 hours	29	8.1	-	-
Total	359	100.0	-	-
Waiting time for examinations and/or tests			−0.31	−0.33
No waiting period	19	6.2	-	-
Up to 15 minutes	57	18.7	-	-
Over 15 and up to 30 minutes	77	25.2	-	-
Over 30 minutes up to 1 hour	56	18.4	-	-
Over 1 and up to 2 hours	48	15.7	-	-
Over 2 and up to 4 hours	35	11.5	-	-
Over 4 and up to 6 hours	11	3.6	-	-
Over 6 and up to 9 hours	2	.7	-	-
Total	305	100.0	-	-
Waiting time to be called back by the doctor after the examinations and/or tests			−0.33	−0.34
No waiting period	15	5.1	-	-
Up to 15 minutes	30	10.1	-	-
Over 15 and up to 30 minutes	39	13.1	-	-
Over 30 minutes up to 1 hour	61	20.5	-	-
Over 1 and up to 2 hours	69	23.2	-	-
Over 2 and up to 4 hours	53	17.8	-	-
Over 4 and up to 6 hours	22	7.4	-	-
Over 6 and up to 9 hours	8	2.7	-	-
Total	297	100.0	-	-
Waiting time from when the patient was informed about discharge until the patient left the hospital			−0.18	−0.17
No waiting period	98	30.9	-	-
Up to 5 minutes	42	13.2	-	-
Over 5 and up to 15 minutes	68	21.5	-	-
Over 15 and up to 30 minutes	38	12.0	-	-
Over 30 minutes and up to 1 hour	34	10.7	-	-
Over 1 hour	37	11.7	-	-
Total	317	100.0	-	-

**Table 3:** Multiple regression analysis results for satisfaction.

	Stand. Beta	T	Sig.
Constant		−1.30	0.20
Global perceptions			
Accessibility and availability	0.07	1.53	0.13
Facilities and physical conditions	0.04	0.69	0.49
Privacy	0.04	0.88	0.38
Busyness of the ED in terms of number of people	0.00	−0.03	0.98
Perceived quality dimensions			
ED personnel			
Staff at the registration counter	0.08	1.60	0.11
Staff conducting the triage	−0.04	−0.95	0.34
Doctors	0.14	3.09	0.00
Health technicians	0.00	−0.08	0.94
Lack of any type of staff	−0.02	−0.50	0.62
Admission to the ED/triage process			
Information about possible delays	0.06	1.82	0.07
Agreement with triage color assigned	0.01	0.39	0.70
Waiting time			
Waiting time for triage (perception)	0.08	2.08	0.04
Waiting time after triage (perception)	0.07	1.55	0.12
Waiting time for examinations and/or tests (perception)	0.02	0.46	0.65
Waiting time to be called back by the doctor (perception)	0.00	−0.04	0.97
Waiting time from when the patient was informed about discharge until the patient left the hospital (perception)	0.03	0.88	0.38
Expectations			
Meeting expectations	0.53	11.44	0.00
Social and demographic attribute:			
Age	0.04	1.18	0.24

*ED*, emergency department.

**Table 4:** Multiple regression analysis results for Perceived Quality of Healthcare.

	Stand. Beta	t	Sig.
Constant		0.36	0.72
Global perceptions			
Accessibility and availability	0.09	1.68	0.09
Facilities and physical conditions	0.06	1.01	0.31
Privacy	0.09	1.89	0.06
Busyness of the ED in terms of number of people	−0.01	−0.35	0.73
Perceived quality dimensions			
ED personnel			
Staff at the registration counter	−0.06	−1.16	0.25
Staff conducting the triage	0.07	1.39	0.17
Doctors	0.43	8.35	0.00
Health technicians	0.00	0.02	0.98
Lack of any type of staff	0.01	0.22	0.83
Admission to the ED/Triage process			
Information about possible delays	0.03	0.77	0.44
Waiting time			
Waiting time for triage (perception)	0.00	0.09	0.93
Waiting time after triage (perception)	−0.06	−1.18	0.24
Waiting time for examinations and/or tests (perception)	0.03	0.58	0.57
Waiting time to be called back by the doctor (perception)	0.10	1.79	0.07
Waiting time from when the patient was informed about discharge until the patient left the hospital (perception)	−0.01	−0.21	0.83
Discharge process			
If the patient was given discharge note	−0.02	−0.65	0.52
Expectations			
Meeting expectations	0.26	5.07	0.00
Social and demographic attribute:			
Age	0.06	1.69	0.09

*ED*, emergency department.
